# Long-term cardiovascular morbidity following hyperemesis gravidarum: A Norwegian nationwide cohort study

**DOI:** 10.1371/journal.pone.0218051

**Published:** 2019-06-12

**Authors:** Stine Fossum, Øyvind Næss, Sigrun Halvorsen, Grethe S. Tell, Åse V. Vikanes

**Affiliations:** 1 Department of Cardiology, Oslo University Hospital, Oslo, Norway; 2 Faculty of Medicine, University of Oslo, Oslo, Norway; 3 Division for Mental and Physical Health, National Institute of Public Health, Oslo, Norway; 4 Department of Global Public Health and Primary Care, University of Bergen, Bergen, Norway; 5 The Intervention Center, Oslo University Hospital, Oslo, Norway; 6 Institute for Clinical Medicine, University of Oslo, Oslo, Norway; University of Cambridge, UNITED KINGDOM

## Abstract

**Objective:**

To investigate whether exposure to hyperemesis gravidarum (hyperemesis) is associated with subsequent maternal cardiovascular morbidity.

**Design:**

Nationwide cohort study.

**Setting:**

Medical Birth Registry of Norway (1967–2002) linked to the nationwide Cardiovascular Disease in Norway project 1994–2009 (CVDNOR) and the Cause of Death Registry.

**Population:**

Women in Norway with singleton births from 1967 to 2002, with and without hyperemesis, were followed up with respect to cardiovascular outcomes from 1994 to 2009.

**Methods:**

Cox proportional hazards regression model was applied to estimate hazard ratios (HRs) with 95% confidence interval (CI).

**Main outcome measures:**

The first hospitalisation due to nonfatal stroke, myocardial infarction or angina pectoris, or cardiovascular death.

**Results:**

Among 989 473 women with singleton births, 13 212 (1.3%) suffered from hyperemesis. During follow-up, a total of 43 482 (4.4%) women experienced a cardiovascular event. No association was found between hyperemesis and the risk of a fatal or nonfatal cardiovascular event (adjusted HR 1.08; 95% CI 0.99–1.18). Women with hyperemesis had higher risk of hospitalisation due to angina pectoris (adjusted HR 1.28; 95% CI 1.15–1.44). The risk of cardiovascular death was lower among hyperemetic women in age-adjusted analysis (HR 0.73; 95% CI 0.59–0.91), but the association was no longer significant when adjusting for possible confounders.

**Conclusion:**

Women with a history of hyperemesis did not have increased risk of a cardiovascular event (nonfatal myocardial infarction or stroke, angina pectoris or cardiovascular death) compared to women without.

## Introduction

Both the European and American guidelines for prevention of cardiovascular disease (CVD) in women now include pregnancy-related complications, such as preeclampsia and pregnancy-induced hypertension, as risk factors [1,2]. CVD is the leading cause of death in women [2,3] and early detection of individuals at risk may prevent major cardiovascular events. Pregnancy-related risk factors for CVD provide such an opportunity.

Hyperemesis gravidarum (hyperemesis), characterized by extreme nausea and vomiting in early pregnancy, is the most common reason for hospitalisation in the first trimester of pregnancy and is associated with several risk factors for CVD [4,5]. These include low socioeconomic status, hypertension, hypercholesterolemia, overweight, autoimmune diseases such as rheumatoid arthritis and diabetes mellitus [6–9]. Hyperemesis has also been found associated with placental dysfunction disorders, i.e. preeclampsia and placental abruption [10–12], both known risk factors for CVD later in life [13–15]. Whether women with hyperemesis have a subsequent increased risk of cardiovascular events has to our knowledge not yet been studied.

We therefore aimed to investigate the risk of fatal and nonfatal cardiovascular events during long-term follow-up in women with and without a history of hyperemesis.

## Materials and methods

### Study population

**Fig 1:**
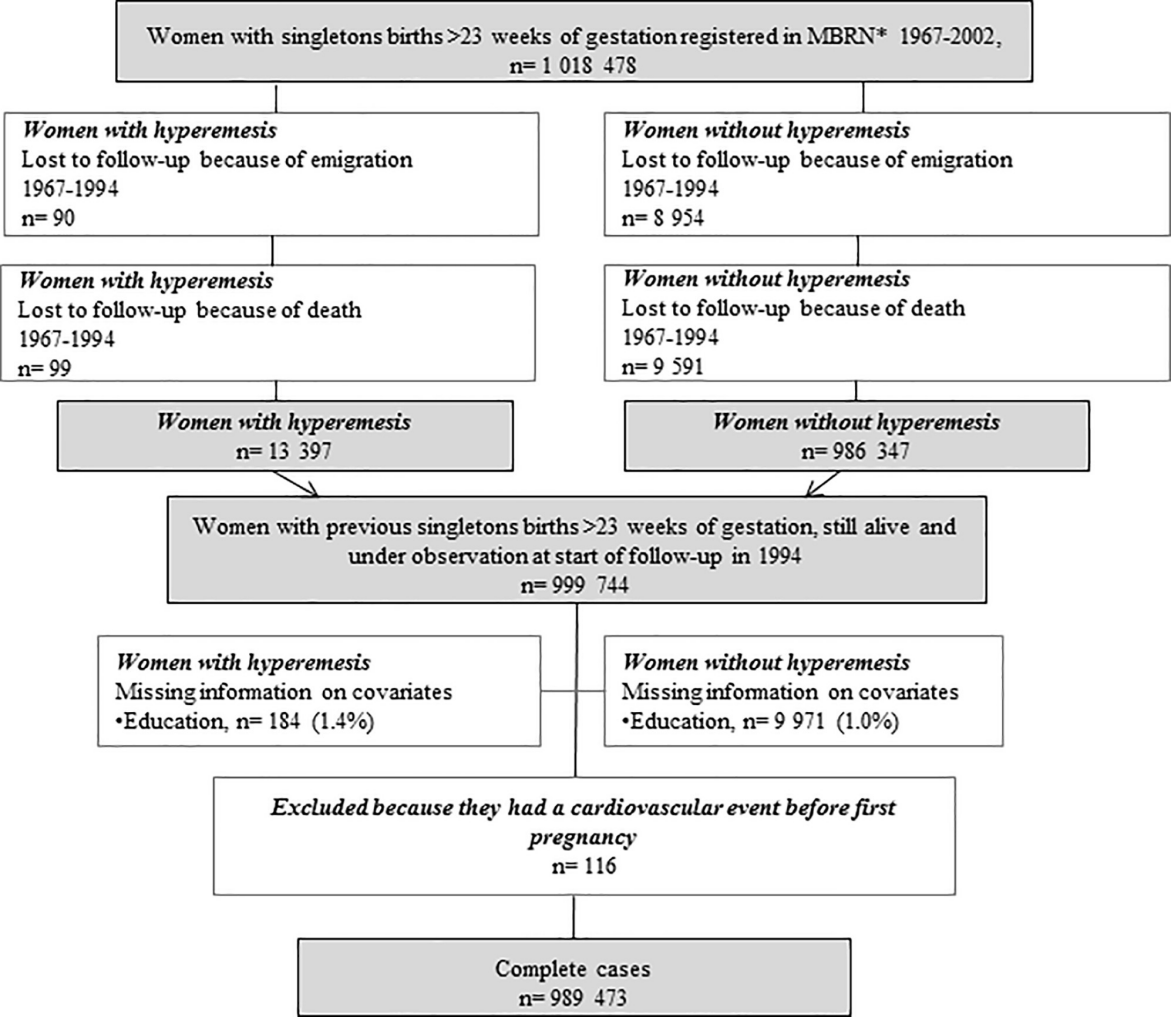
Flow diagram of the study population. Data on 1 018 478 women with registered pregnancies in the Medical Birth Registry of Norway (MBRN) in 1967–2002 were available. The figure shows how complete cases at start of follow-up were defined.

From 1967 to 2002, all pregnancies ending after week 16 were registered in the Medical Birth Registry of Norway (MBRN) [16]. This registration is mandatory and has to be done within one week after discharge from the delivery unit. Information on maternal health before and during pregnancy, complications during pregnancy and delivery as well as information about the infant are registered. The study population comprised women with singleton births of more than 23 weeks of gestation registered in the MBRN during 1967–2002, being alive in Norway at the start of follow-up (Fig 1).

### Exposure

From 1967 to 1998, pregnancy complications were reported in the MBRN in free text according to the International Classification of Disease (ICD). Women with hyperemesis were registered with ICD-8 codes 638.0 (hyperemesis gravidarum with neuritis) and 638.9 (hyperemesis gravidarum without mention of neuritis) until 1998, and from 1999 and onwards hyperemesis was registered by the ICD-10 codes O21.0 (mild hyperemesis gravidarum), O21.1 (hyperemesis gravidarum with metabolic disturbances) and O21.9 (vomiting in pregnancy, unspecified) [17].

### Follow-up

By using the personal identification number unique to every Norwegian resident, data from the MBRN were linked to the Cause of Death Registry and hospital discharge data on cardiovascular events obtained from the Cardiovascular Disease in Norway project (CVDNOR). In CVDNOR (https://cvdnor.b.uib.no/), all hospitalisations due to CVD or diabetes mellitus have been collected from all Norwegian somatic hospitals from 1994 through 2009 (1994 was the first year all hospitals in Norway started to use electronic patient administrative systems). CVDNOR has been described in detail elsewhere [18,19]. Information on death due to CVD during the same time period was obtained from the Norwegian Cause of Death Registry, which has a 98% coverage and completeness of the Norwegian population. For all deaths, a death certificate must be completed by a physician. A code from the ICD system is allocated to the diagnoses in the death certificate [20]. The registry used ICD-9 from 1986 to 1995 and ICD-10 codes from 1996 to 2009. Women with singleton births registered in the MBRN during 1967–1994 were followed with respect to cardiovascular outcomes from 1994 through 2009. Women with singleton births registered in the MBRN during 1994–2002 were followed through 2009.

### Outcome

The primary outcome was defined as the occurrence of cardiovascular death, nonfatal myocardial infarction (I21, I22), nonfatal stroke (I60-I61, I63-I64) or hospitalisation due to angina pectoris (I20, I25.1) as main or secondary discharge diagnosis in a time-to-event analysis. Cardiovascular death was defined as CVD (I00-I99) as the underlying cause of death registered in the Cause of Death Registry or death within 28 days after hospitalisation with a cardiovascular event (I00-I99). Secondary outcome was defined as the primary outcome, excluding angina pectoris. In addition, separate analyses for each component of the primary outcome were conducted.

### Covariates

Age at first birth was the woman’s age at her first registered birth in the MBRN. Since some women delivered children before 1967, a parity-variable reflecting the mother’s self-reported parity was used. Information on maternal country of origin was provided from Statistics Norway.

Information on gestational hypertension, placental abruption, pre-gestational hypertension and pre-gestational diabetes mellitus was obtained from the MBRN. Based on information from each woman’s registered pregnancies, dichotomous variables were created (never/ever). Information on smoking and maternal body weight was not available.

Information on maternal highest education at the end of follow-up was obtained from Statistics Norway and categorized as basic (9 years (7 years in the 1960s)), secondary (10–12 years) or tertiary (≥13 years), according to the Norwegian Standard Classification of Education [21].

### Statistical methods

The analyses were conducted in STATA version 15. Descriptive statistics of women with and without hyperemesis are presented as median (25 and 75 percentiles) or as numbers (%). Cox proportional hazards regression model was applied to estimate hazard ratios (HRs) for time-to-event outcomes. Women with previous births, still alive and living in Norway at start of follow-up were followed from 1994 until a CVD event occurred or censored if dead from other causes, emigration or at the cut-off date of December 31^st^ 2009, whichever occurred first. Since angina as a discharge diagnosis may be more prone to bias, we also performed the analyses without angina as a secondary outcome. In addition, the occurrence of a nonfatal myocardial infarction, nonfatal stroke, angina pectoris or cardiovascular death were assessed individually regardless of the order of which the events occurred if a woman had experienced more than one event during follow-up. The time variable in the Cox-models was “years from 1994 (or first pregnancy if later than 1994) to the event of interest/censored”. In addition to the crude analyses, age-adjusted (Model 1) and multivariable-adjusted (Model 2) analyses were performed. Based on prior knowledge [4,6,12,22], the following covariates were considered associated with both hyperemesis and the studied outcome, and were included as potential confounders: the woman’s age at first pregnancy and year of birth, highest obtained education, country of birth, hypertensive disorders in pregnancy, placental abruption, pre-gestational diabetes and pre-gestational hypertension. Less than 1.5% of the women had missing information on education, information on the other covariates were complete. An estimate with a confidence-interval without one or a *p-*value below 0.05 was considered statistically significant.

Ethical approval for the study was obtained from the Regional Committee for Medical and Health Research Ethics (2015/1347/REK South-East). Due to the large number of women registered in the MBRN and the CVDNOR project, the ethical committee approved the study, making an exception from the general rule of necessitating consent from all women included. The data was de-identified to preserve the participants’ privacy.

### Additional analyses

The primary outcome was also assessed in a subgroup of women born before 1945. This group included the oldest women in the study population, aged 50 years or older at start of follow-up. This was done to investigate if the studied associations differed according to if only women at the highest risk of a cardiovascular event in the population were included.

## Results

Among 1 018 478 women with singleton births during 1967–2002, 9 044 (0.9%) emigrated and 9 690 (1.0%) died before start of follow-up. Less than 1.5% had missing information on covariates and only complete cases were used for analyses (Fig 1). The study sample comprised 989 473 women, of which 13 212 (1.3%) had suffered from hyperemesis in at least one pregnancy. The median follow-up time was 15 years (range 0–15) and total person-years at risk were 13 527 714. Lost to follow-up because of emigration was 10 360 (1.1%) women and 20 719 (2.1%) women were censored due to death from other causes during follow-up (1994–2009). Women with a history of hyperemesis were younger at their first registered pregnancy and were less often of ethnic Norwegian origin compared to women without hyperemesis. There was no difference between the two exposure groups in the proportion of women with pre-gestational diabetes mellitus or pre-gestational hypertension. Women with a history of hyperemesis were younger at start of follow-up. At the end of follow-up, women with previous hyperemesis were younger, had obtained a higher level of education and were more often multipara, compared to women without hyperemesis (Table 1).

**Table 1:** Characteristics of the study cohort: Women in Norway with singleton births from 1967 to 2002 (n = 989 473).

Maternal and pregnancy characteristics	Women with hyperemesis gravidarum (n = 13 212)	Women without hyperemesis gravidarum (n = 976 261)	*P-*value**
**At time of delivery**			
Median age at first pregnancy*	24 (21–27)	25 (21–28)	<0.01
Age at first reg. pregnancy, n (%)			<0.01
≤19	1 574 (11.9)	117 031 (12.0)	
20–24	5 677 (43.0)	368 935 (37.8)	
25–29	4 136 (31.3)	304 875 (31.2)	
30–34	1 333 (10.1)	127 826 (13.1)	
≥35	492 (3.7)	57 594 (5.9)	
Pre-gestational diabetes, n (%)	44 (0.3)	3 672 (0.4)	0.4
Pre-gestational hypertension, n (%)	63 (0.5)	4 386 (0.5)	0.6
Maternal country of origin, n (%)			<0.01
Norway	11 565 (87.5)	880 279 (90.2)	
Europe	758 (5.7)	57 747 (5.9)	
Africa	170 (1.3)	4 853 (0.5)	
Asia	507 (3.8)	19 447 (2.0)	
North-America	165 (1.3)	10 931 (1.1)	
South-America	40 (0.3)	2 517 (0.3)	
Oceania	7 (0.1)	487 (0.1)	
**At start of follow-up**			
Median age at start of follow-up*	35 (28–45)	37 (29–46)	<0.01
**At end of follow-up**			
Median age at the end of study*	50 (42–59)	52 (43–61)	<0.01
Min, max age at the end of study	22, 89	19, 91	
Highest obtained education, n (%)			<0.01
Basic	3 367 (25.5)	248 107 (25.4)	
Secondary	5 797 (43.9)	444 304 (45.5)	
Tertiary	4 048 (30.6)	283 850 (29.1)	
Parity by end of follow-up, n (%)			<0.01
Primipara	1 727 (13.1)	201 865 (20.7)	
Multipara	11 485 (86.9)	774 396 (79.3)	
Preeclampsia, pregnancy-related hypertension and eclampsia, n (%)	985 (7.5)	73 581 (7.5)	0.7
Placental abruption, n (%)	169 (1.3)	10 911 (1.1)	0.1

*Median with 25 and 75 percentiles

**Tested with t-test or chi-squared test

### Primary outcome

**Fig 2:**
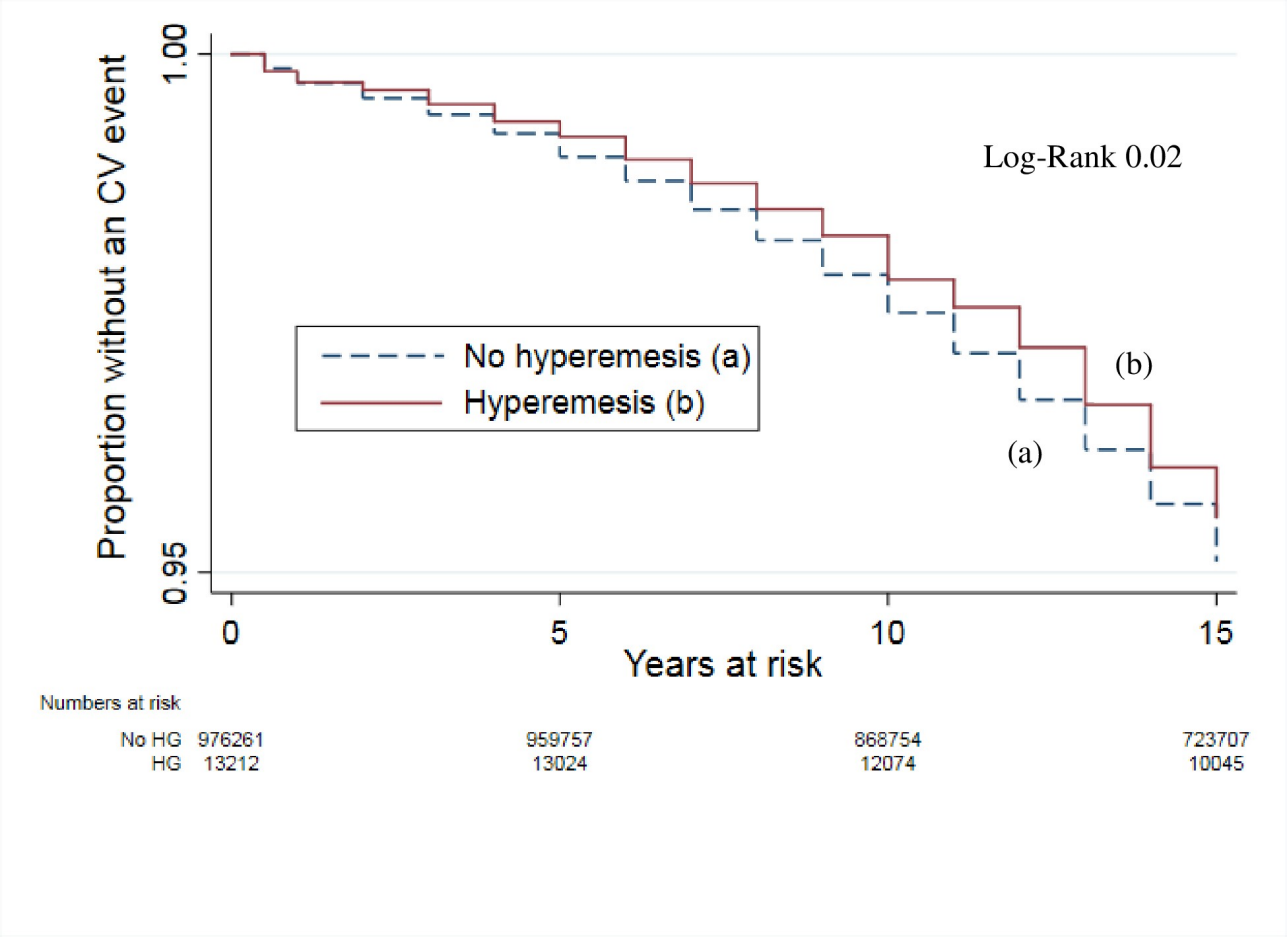
Event-free survival during follow-up (1994–2009). Women in Norway with a history of hyperemesis gravidarum (n = 13 212) compared to women without such history (n = 976 261).

Among women with a history of hyperemesis, 535 (4.1%) experienced at least one cardiovascular event during follow-up, compared to 42 947 (4.4%) of the women without such history (Table 2). In the crude analysis, women with hyperemesis had a lower risk of a cardiovascular event compared to women without such history (Fig 2 and Table 2), but this association was no longer present after adjustment for age and other available confounders (Table 2). When the effect of each confounder was considered individually, we found that the change from Model 1 to Model 2 was mainly driven by the woman’s year of birth.

**Table 2:** Primary and secondary outcomes during 15 years of follow-up (1994–2009) in women with a history of hyperemesis gravidarum (n = 13 212) compared to women without (n = 976 261) in Norway.

Cardiovascular event	Number (%) of women with events according to HG status		Hazard ratio (95% confidence interval) for CVD eventNo HG as referent group		
	HG (n = 13 212)	No HG (n = 976 261)	Crude model	Model 1*	Model 2**
**Primary outcome**					
*CVD death*, *nonfatal MI*, *nonfatal stroke or hospitalization with angina pectoris*	535 (4.1)	42 947 (4.4)	0.90 (0.83–0.98)	0.98 (0.90–1.07)	1.08 (0.99–1.18)
**Main secondary outcome**					
*CVD death*, *nonfatal MI or nonfatal stroke*	319 (2.4)	29 033 (3.0)	0.80 (0.71–0.89)	0.88 (0.78–0.98)	0.96 (0.86–1.08)
**Additional secondary outcomes**					
*Death from CVD*	81 (0.6)	9 333 (1.0)	0.63 (0.51–0.78)	0.73 (0.59–0.91)	0.81 (0.65–1.01)
*Hospitalisation with angina*	299 (2.3)	20 151 (2.1)	1.08 (0.96–1.21)	1.16 (1.03–1.30)	1.28 (1.15–1.44)
*Nonfatal MI*	126 (1.0)	11 063 (1.1)	0.83 (0.69–0.99)	0.90 (0.76–1.08)	1.01 (0.84–1.20)
*Nonfatal stroke*	163 (1.2)	13 038 (1.3)	0.91 (0.78–1.06)	0.99 (0.85–1.15)	1.07 (0.92–1.25)

HG: hyperemesis gravidarum, CVD: cardiovascular disease, MI: myocardial infarction

*Age-adjusted

**Adjusted for woman’s age at first birth, woman’s year of birth (categorical), country of birth, education, hypertensive disorder in pregnancy, pre-gestational hypertension, pre-gestational diabetes, placental abruption.

### Secondary outcomes

After excluding angina as a part of the composite outcome, 319 (2.4%) of the women with a history of hyperemesis had experienced a cardiovascular event (cardiovascular death, nonfatal myocardial infarction or stroke) during follow-up, compared to 29 033 (3.0%) of the women without such history. In the crude analysis there was a lower risk of a cardiovascular event among women with a history of hyperemesis compared to women without hyperemesis, and still significantly lower after adjustment for age, but after adjustment for other available confounders, the association was no longer significant (Table 2).

During follow-up, women with a history of hyperemesis had lower risk of cardiovascular death compared to women without such history (crude HR 0.63; 95% CI 0.51–0.78) (Table 2). The association was still significantly lower after age-adjustment, but after adjustment for other available confounders, the association was no longer significant. The risk of nonfatal myocardial infarction or stroke did not differ according to hyperemesis-status in pregnancy (Table 2). Women with hyperemesis had a higher risk of being hospitalised with angina pectoris, both in the age-adjusted and multivariable-adjusted model (Table 2).

### Additional analyses

Among 165 327 women born before 1945 with a previous pregnancy, 1743 women had suffered from hyperemesis. During 15 years of follow-up, a total of 23 287 (14.1%) women experienced a cardiovascular event (primary outcome). In the subgroup of older women, those with a history of hyperemesis had similar risk of a cardiovascular event as women without (Table 3).

**Table 3:** Primary outcome during 15 years of follow-up (1994–2009) in women born before 1945 with a history of hyperemesis gravidarum (n = 1 743) compared to women without (n = 163 584) in Norway.

Cardiovascular event	Number (%) of women with events according to HG status		Hazard ratio (95% confidence interval) for CVD eventNo HG as referent group		
	HG (n = 1 743)	No HG (n = 163 584)	Crude model	Model 1*	Model 2**
**Primary outcome** *First hospitalisation with MI*, *stroke or angina pectoris*, *or CVD death*	242 (13.9)	23 045 (14.1)	0.98 (0.86–1.11)	1.06 (0.94–1.21)	1.08 (0.95–1.23)

HG: hyperemesis gravidarum, CVD: cardiovascular disease, MI: myocardial infarction

*Age-adjusted

**Adjusted for woman’s age at first birth, woman’s year of birth (categorical), country of birth, education, hypertensive disorder in pregnancy, pre-gestational hypertension, pre-gestational diabetes, placental abruption.

## Discussion

### Main findings

In this large nationwide cohort study, we found no evidence of increased risk of a cardiovascular event (nonfatal myocardial infarction or stroke, angina pectoris or cardiovascular death) long-term in women with hyperemesis compared to those without.

### Strengths and limitations

A major strength in this study is the large nationwide study population and the long follow-up-time for cardiovascular events. The MBRN and the Cause of Death Registry have mandatory reporting, and CVDNOR contains information on CVD hospitalisations from all somatic hospitals in Norway in the time-period. Moreover, the linkage of the MBRN to both CVDNOR and the Cause of Death Registry made it possible to include cardiovascular deaths outside hospital and increase the accuracy by defining cardiovascular death as either death within 28 days after discharge with a cardiovascular event or CVD as the underlying cause of death on the death certificate.

The change in estimate from Model 1 to Model 2 was mainly due to the adjustment for maternal year of birth. This change was also found independent of adjustment for age. We assessed the difference in effect estimates in different birth cohorts and found slightly different effect estimates in different strata, but all the HRs pointed to the same overall result with estimates close to one and negative findings. The small change in estimate may be the consequence of a cohort effect [23] because of heterogeneity in follow-up time for events between young and old segments of the population. The lack of information on cardiovascular events in the period before 1994 is another limitation. On the other hand, cardiovascular events in women are most likely to occur after the age of 50 [19,24] and 90% of the women in this study were younger than 53 years at start of follow-up in 1994, making them less likely to have suffered from a cardiovascular event before follow-up started. Moreover, the uncertainty related to angina as a discharge diagnosis may have led to inclusion of events representing non-cardiac chest pain [25]. It is therefore not known whether the increased risk of being hospitalised due to angina pectoris among women with previous hyperemesis indicates an increased risk of later ischemic heart disease or not.

Although incorrect registration is a limitation in all register-based research, hyperemesis in the MBRN has previously been validated and found eligible for large-scale epidemiological studies [17]. Moreover, the MBRN did not contain information on potential confounders, such as smoking-habits or body mass index before 1999 and 2006, respectively. Smoking is associated with a reduced risk of hyperemesis and hyperemesis is associated with both underweight and obesity [7]. We also lacked information on hypertension, diabetes and cholesterol at start of follow-up. The lack of potential confounder control may have contributed to residual confounding. However, we have previously shown that hyperemetic women at the age of 40 have similar cardiovascular risk factor profiles as women without hyperemesis [26].

### Comparison with other studies

Few previous studies have explored cardiovascular risk subsequent to hyperemesis. Some large population-based studies have, however, found women with a history of hyperemesis to have increased risk of preeclampsia [10,12] and autoimmune diseases such as rheumatoid arthritis [9,27]. Immunological abnormalities and increase of fetal cells in maternal circulation may reflect possible underlying mechanisms, such as abnormal placentation and increased levels of human chorionic gonadotropin (hCG) [10]. Such mechanisms could also contribute to explain associations between autoimmune disease and hyperemesis.

Hyperemesis during second trimester is found to be strongly associated with preterm pre-eclampsia, placental abruption as well as a giving birth to a small-for-gestational-age baby [12]. Despite the fact that all aforementioned conditions are associated with increased risk of CVD later in life [13,28], we did not find any evidence of increased risk for cardiovascular events subsequent to hyperemesis. This is, however, in line with findings in our previous articles on midlife cardiovascular risk factors subsequent to hyperemesis, and on risk of cardiovascular death among women with a history of hyperemesis [26, 29]. Compared to our previous paper on long-term mortality following hyperemesis, the slightly lower HR for cardiovascular death in crude and age-adjusted analyses in the present study may be explained by different follow-up time and a broader definition of cardiovascular death. In the previous paper cardiovascular death was defined as CVD as the underlying cause of death registered in the Cause of Death Registry [29]. In the present paper, we defined cardiovascular death as CVD as the underlying cause of death registered in the Cause of Death Registry or death within 28 days after hospitalisation with a cardiovascular event.

### Interpretation

Results of the current study indicate that women with a history of hyperemesis do not have higher risk of cardiovascular events later in life, indicating that they may have the same cardiovascular follow-up as the female population in general.

Although the study population was relatively young at the end of follow-up, 25% of the women were above 60 years and it is unlikely that hyperemesis is associated with increased risk of a premature cardiovascular event. This assumption is furthermore supported by the large cohort, number of events and long follow-up time. Additional analyses on women aged 50 years or older at start of follow-up revealed no increase in risk of a cardiovascular event among women with a history of hyperemesis compared to women without. When conducting sub-analyses, exploring each cardiovascular event separately, we found that hyperemetic women had slightly increased risk of being hospitalised due to angina pectoris. The difference was significant in the adjusted model only, something which makes the interpretation difficult. Moreover, the diagnostic criteria for myocardial infarction have changed over time, and troponins were first introduced in Norwegian hospitals in 1999–2001 [30]. This means that women previously diagnosed with angina, may after introduction of troponins have been diagnosed with a myocardial infarction. This would, however, probably not have changed the results for the primary outcome. It is not known whether women with angina in our study have suffered from a myocardial infarction after follow-up and because of the relatively young population, this could be a topic for future research.

## Conclusion

In this large nationwide cohort study, we found no evidence of increased risk of a cardiovascular event (nonfatal myocardial infarction or stroke, angina pectoris or cardiovascular death) in women with a history of hyperemesis compared to women without.
